# From GenBank to GBIF: Phylogeny-Based Predictive Niche Modeling Tests Accuracy of Taxonomic Identifications in Large Occurrence Data Repositories

**DOI:** 10.1371/journal.pone.0151232

**Published:** 2016-03-11

**Authors:** B. Eugene Smith, Mark K. Johnston, Robert Lücking

**Affiliations:** 1 Integrative Research Center & Gantz Family Collections Center, Science & Education, The Field Museum, 1400 South Lake Shore Drive, Chicago, Illinois, 60605–2496, United States of America; 2 Science Action Center, Science & Education, The Field Museum, 1400 South Lake Shore Drive, Chicago, Illinois, 60605–2496, United States of America; 3 Botanical Garden and Botanical Museum, Königin-Luise-Str. 6–8, 14195, Berlin, Germany; Trier University, GERMANY

## Abstract

Accuracy of taxonomic identifications is crucial to data quality in online repositories of species occurrence data, such as the Global Biodiversity Information Facility (GBIF), which have accumulated several hundred million records over the past 15 years. These data serve as basis for large scale analyses of macroecological and biogeographic patterns and to document environmental changes over time. However, taxonomic identifications are often unreliable, especially for non-vascular plants and fungi including lichens, which may lack critical revisions of voucher specimens. Due to the scale of the problem, restudy of millions of collections is unrealistic and other strategies are needed. Here we propose to use verified, georeferenced occurrence data of a given species to apply predictive niche modeling that can then be used to evaluate unverified occurrences of that species. Selecting the charismatic lichen fungus, *Usnea longissima*, as a case study, we used georeferenced occurrence records based on sequenced specimens to model its predicted niche. Our results suggest that the target species is largely restricted to a narrow range of boreal and temperate forest in the Northern Hemisphere and that occurrence records in GBIF from tropical regions and the Southern Hemisphere do not represent this taxon, a prediction tested by comparison with taxonomic revisions of *Usnea* for these regions. As a novel approach, we employed Principal Component Analysis on the environmental grid data used for predictive modeling to visualize potential ecogeographical barriers for the target species; we found that tropical regions conform a strong barrier, explaining why potential niches in the Southern Hemisphere were not colonized by *Usnea longissima* and instead by morphologically similar species. This approach is an example of how data from two of the most important biodiversity repositories, GenBank and GBIF, can be effectively combined to remotely address the problem of inaccuracy of taxonomic identifications in occurrence data repositories and to provide a filtering mechanism which can considerably reduce the number of voucher specimens that need critical revision, in this case from 4,672 to about 100.

## Introduction

Accuracy of taxonomic identifications of vouchers is a critical element of data quality in biodiversity repositories. While users tend to assume that the underlying taxonomy is correct, specialists are aware that a substantial proportion of vouchers might be wrongly identified, caused by lack of expertise of the identifier or by inappropriate species concepts [1–13]. The charismatic lichen fungus, *Usnea longissima* Ach., Methuselah's Beard Lichen, a member of the megadiverse macrolichen family Parmeliaceae, with its showy, long-pendulous thalli covering tree branches like garlands, is well-known even to amateurs and frequently collected or reported. Misidentifications with other long-pendulous species of *Usnea* are common, although the obvious differences have been worked out in taxonomic treatments [14–25]. In addition, lichens of other genera, such as *Ramalina usnea*, and even non-lichens such as the common, pendulous bromeliad *Tillandsia usneoides*, are not rarely mistaken for *U*. *longissima*. Such misidentifications are not trivial, since accurate taxonomy is crucial for studies on the ecological importance of species and their potential uses: in the case of *U*. *longissima*, this species is used as an indicator of well-conserved, northern-temperate and boreal forest ecosystems [25–36].

The potential magnitude of the problem becomes obvious when looking at the most important public biodiversity repositories. Over the past 25 years, nearly 180 million sequences were deposited in GenBank and over 200 million in the Whole Genome Shotgun (WGS) database, as well as 1.7 quadrillion (1.7 × 10^15^) open access bases, corresponding to roughly 3.5 trillion (3.5 × 10^12^) sequence reads, in The NCBI Sequence Read Archive (SRA) [37–40]. GenBank sequences are directly linked to taxonomic identifications and, through barcoding initiatives, serve as direct reference for identification purposes [41–47]. However, especially for fungi, including lichens, the accuracy of sequence identifications has been questioned, and about 20% of sequence entries, including approximately 700,000 ITS barcoding sequences, have been estimated to be incorrectly labeled [1, 4, 8, 12–13]. A solution to this are curated ITS databases [1, 48–49].

About 15 years ago, efforts began to make specimen occurrence data from natural history collections broadly available through online data repositories [50–53]. By far the largest is the Global Biodiversity Information Facility (GBIF), set up by the Organization for Economic Cooperation and Development (OECD), which currently includes 526 million occurrence records, more than DNA sequences available through GenBank and the WGS together, and including nearly 10 million fungal and lichen occurrences [54]. The widely used Consortium of North American Lichen Herbaria and Bryophyte Herbaria (CNALH, CNABH), based on the Symbiota platform, currently host over 4 million records [55–56]. A massive effort to digitize North American natural history collections is being funneled through the iDigBio specimen portal, which has accumulated more than 25 million records, including over 2.5 million fungi and lichens [57]. However, even more so than DNA sequence data, occurrence records are often unreliable, due to incorrect specimen identifications and lack of taxonomic revision especially of historical collections [5–6, 7, 10–12]. Among fungi and lichens, depending on the group under study, up to 50% of occurrence data may have incorrect taxonomic labels [8].

As important biodiversity resources, both sequence and occurrence data rely on voucher specimens and are affected by potentially inaccurate identifications [49, 58]. However, while DNA sequence data provide intrinsic information as to their correct placement and wrongly identified entries are readily detected [8, 48], this is not possible for occurrence data, unless accompanied by high quality specimen images, which allow for remote taxonomic assessment, such as type specimens digitized through the Global Plants Initiative [59]. Unfortunately, taxonomic revision of millions of specimens is virtually impossible, not just due to the taxonomic impediment, the dwindling support for taxonomic studies and the resulting loss of expertise [60–67], but simply because of the magnitude of the problem. Even if taxonomy were alive and well, thousands of experts would be needed full-time to provide correct identifications for millions of specimens within a reasonable time frame. One way to address this problem is a scoring system that attaches quality scores to occurrence data based on label information, including the taxonomic expertise of the annotator or published citations of the specimen and links to DNA sequences [12, 68]. Unfortunately, such information is usually not available.

Here, we present a different strategy, which combines DNA sequence data and specimen occurrence data to potentially identify incorrectly identified specimens in large repositories such as GBIF. The method applies predictive niche modeling [69–70] to georeferenced specimen data that at the same time have been confirmed to represent a single species by DNA sequence data. As case study, we use *Usnea longissima*, not only because of its enigmatic status, but because it is one of the few species for which georeferenced sequence data are currently available [17]. Indeed, only few fungi and lichens have been studied using predictive niche modeling [71–72]. We tested our method by comparing GBIF occurrence records falling outside the predicted niche with monographic treatments of the genus *Usnea* in the regions in question. To that end, we developed a novel PCA ordination approach to delimit the predicted realized niche within the theoretical niche. This method is a promising tool to address data quality in specimen occurrence data repositories by filtering and returning a small set of specimens that should be focused upon for critical taxonomic revision. The approach can be used for any taxon, as long as sequence data are available to allow for establishment of a statistically supported species concept and the underlying vouchers are (or can be) georeferenced.

## Results

### Phylogenetic Analysis

**Fig 1:**
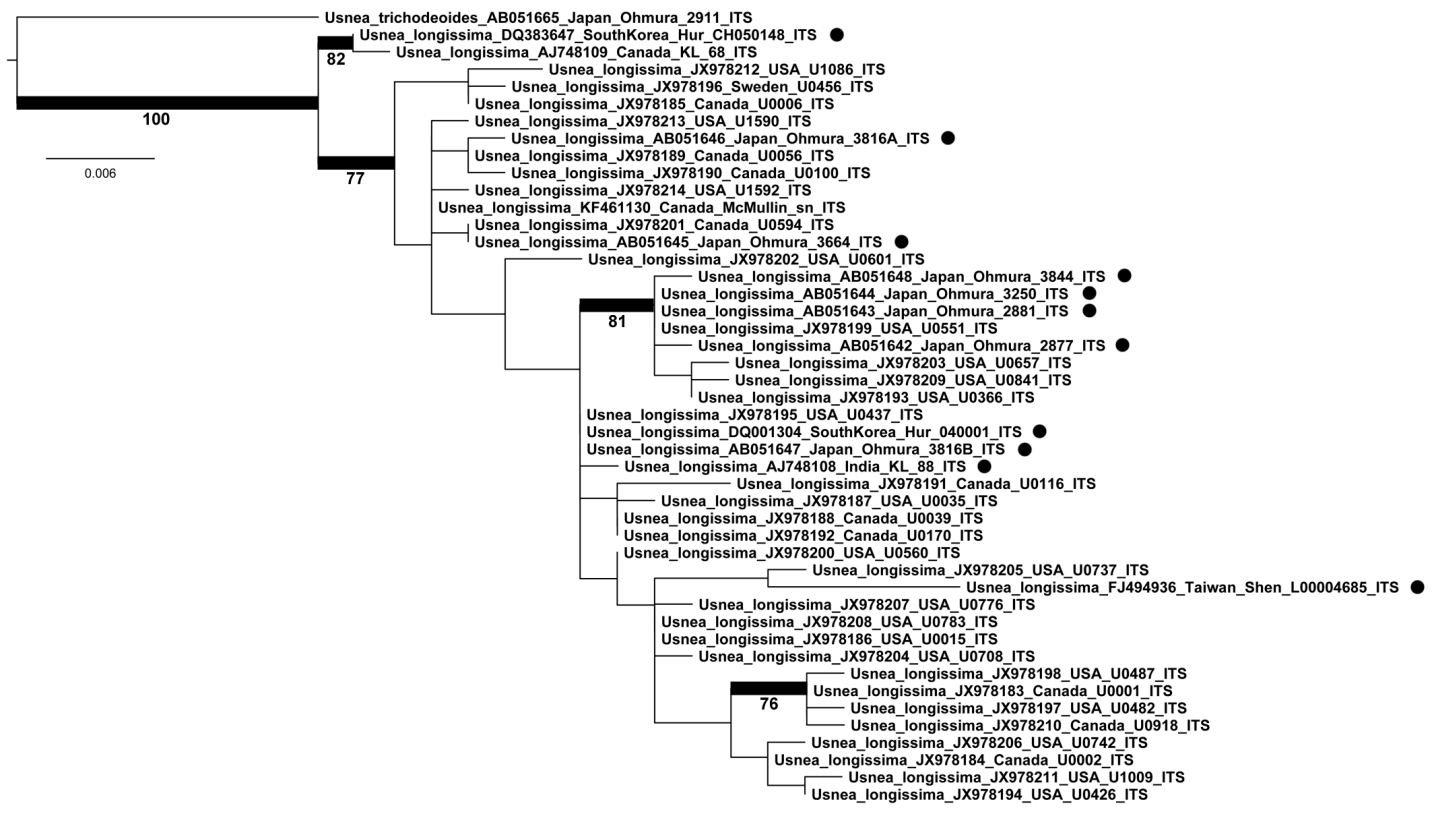
Maximum likelihood tree of *Usnea longissima* haplotypes using the fungal ITS barcoding marker. Bootstrap values are given for supported branches (> 70). Black dots indicate Asian voucher samples (all others North America and one from Europe). Scale bar indicates rate of changes per site. The ingroup sequences of the JX978-series are individual haplotypes representing a total of 1477 sequenced specimens, with each sequence representing a selected specimen corresponding to that particular haplotype.

In accordance with previous studies [23, 24], based on maximum likelihood analysis of 46 ITS barcode sequences, including all individual haplotypes corresponding to 1,477 sequenced samples from 160 georeferenced localities [17], *Usnea longissima* forms a monophyletic clade sister to *U*. *trichodeoides*, with two sequences from Canada and South Corea being supported sister to all other haplotypes (Fig 1). There was no distinct geographic signal in the main clade, with haplotypes from North America, Europe, and Asia mixed in several subclades.

### Predictive Niche Modeling

**Fig 2:**
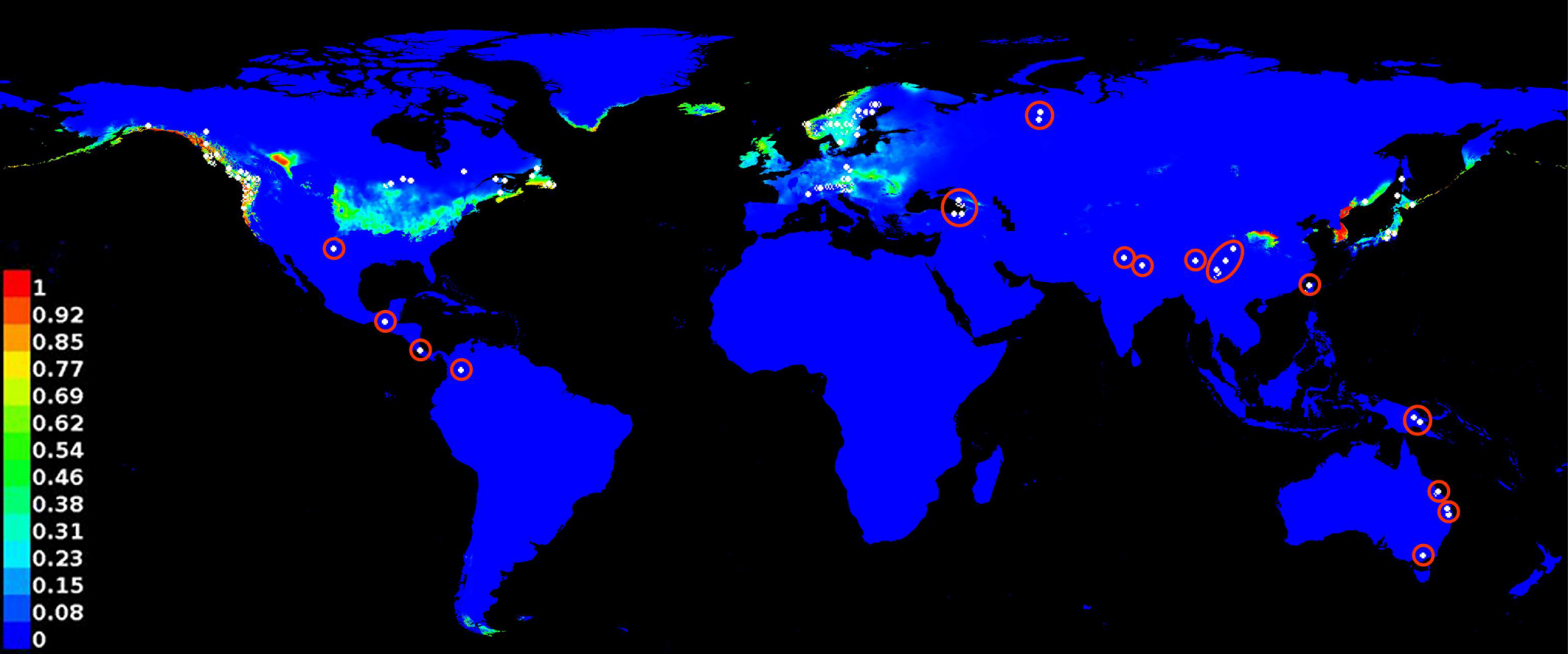
Best fitting MexEnt model for *Usnea longissima* based on 1477 sequenced samples corresponding to 160 localities from Rolstad et al. (2013), indicated by shaded areas ranging from pale blue-green to red. Bright blue areas indicate range of predicted absence. Map is overlayed by occurrence records from GBIF (white dots), and those falling outside the predicted range are marked with red circles. One dot may include more than one GBIF record (S1 Table).

The best fitting MaxEnt model was based on 160 georeferenced localities, which represent a total of 1,477 sequenced specimens of *Usnea longissima* [17]. After correction for sampling bias (see Methods), it predicts the potential niche for *Usnea longissima* along the coast in the Pacific Northwest of North America (California to Alaska), along the eastern US-Canadian border (Great Lakes area) and the eastern Canadian border, Iceland, the British Isles, eastern Scandinavia, and the Alps, a small area in China south of Mongolia, and the Asian east coast (including South and North Corea and Russia) and Japan, as well as southern Patagonia (Fig 2).

Of 3,950 georeferenced GBIF occurrence records of *Usnea longissima* analyzed, 291 fit the predicted range at an AUC (Area Under Curve; see Methods) score of 0.90 or higher (with the highest value = 0.97931 found for 143 occurrence records, corresponding to 131 unique georeferenced localities), 895 at a score of 0.70 or higher, 2,076 at a score of 0.50 or higher, 2,169 at a score of 0.30 or higher, and 2,349 at a score of 0.10 or higher (S1 Table). Depending on whether the limit is set at 0.30 or 0.10, this means that between 541 and 2,153 occurrence records are outside the predicted range, including from northeastern Canada, New Mexico, Mexico, Costa Rica, Colombia, the eastern Black Sea area, the northwestern border of Siberia, southwestern and central China northeast of Nepal (wider Himalaya region), Papua New Guinea, and eastern Australia (Queensland to Victoria). Thus, the model would specifically identify the latter records for taxonomic scrutiny and revision, and particularly those reported from the tropics and the Southern Hemisphere (Mexico, Costa Rica, Colombia, Papua New Guinea, Australia).

**Fig 3:**
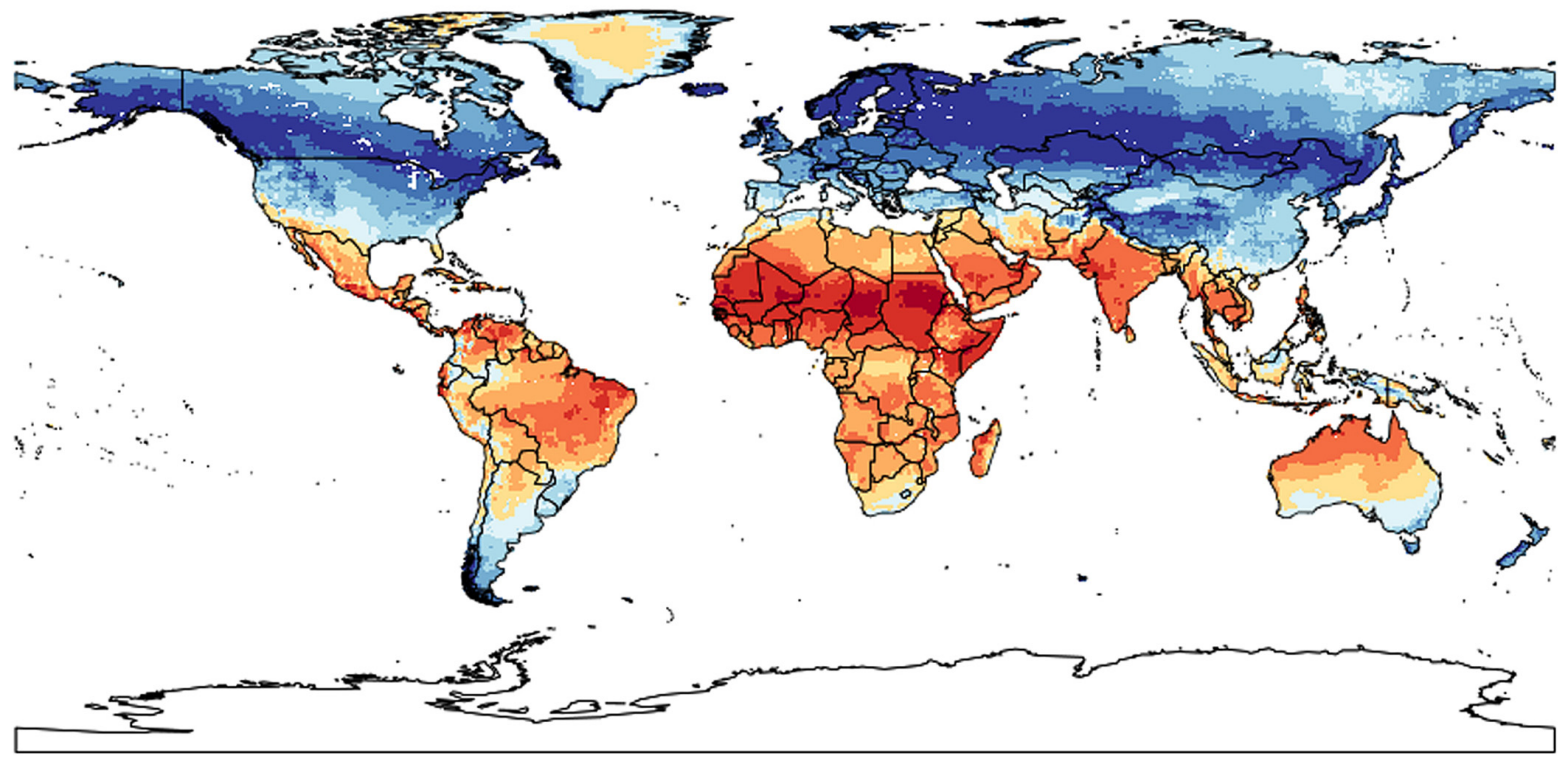
Global mapping of absolute distance scores derived from the first axis of a PCA ordination of environmental grid parameters used for the predictive niche modeling. Distances were computed from an optimal environmental parameter set defined by the highest AUC values for grids with predicted distribution of *Usnea longissima*. Blue areas indicate zero or short ecological distances from the optimal grid whereas red areas indicate far distances (ecogeographical barriers). The tropics emerge as a strong barrier for the north-south distribution of the species.

Principal Component Analysis of the bioclim variables and other environmental grid parameters (see Methods) explained a cumulative variance of 71% on the first two axes, with most variables having high loadings on the first axis (Table 1), which was positively correlated with strong seasonality and negatively with high mean and maximum temperature, whereas the second axis was positively correlated with diurnal temperature range and negatively with high precipitation (Table 1). While these correlations do not reflect the ecological niche of *Usnea longissima*, they determine the internal correlation structure of the underlying bioclim variables. When setting the point on the first axis that reflects the highest AUC score for the occurrence of *Usnea longissima* to zero and transforming the factor scores into absolute distances from that point (see Methods), the tropics are highlighted as a strong ecogeographical barrier for the north-south distribution of the species (Fig 3; S2 Table). This finding indicates that tropical and Southern Hemisphere reports of this species are incorrect, which was subsequently tested by analyzing monographic revisions (including unpublished data) of the genus *Usnea* in the areas where the outliers occurred, in particular Mexico, Costa Rica, Colombia, and Australia (see Discussion). All revisions confirm the absence of *Usnea longissima* in these regions, highlighting common misidentifications with similarly long, pendulous species which, however, differ in branching pattern, surface morphology, internal anatomy, and secondary chemistry [15, 19–21; M. Herrera-Campos, P. Clerc, pers. comm. 2014].

**Table 1:** Factor loadings of the environmental variables used in the PCA and total variance explained. High loadings of >0.70 are highlighted in boldface and marked with an asterisk.

Variable	Factor 1	Factor 2
tree_cover	0.143366	0.441376
alta	0.155203	-0.005793
bio_1a (annual mean temperature)	**-0.932586** (*)	0.333335
bio_2a (mean diurnal temperature range)	-0.333610	**0.711211** (*)
bio_3a (isothermality)	**-0.912183** (*)	0.047748
bio_4a (temperature seasonality)	**0.898851** (*)	0.000346
bio_5a (maximum temperature warmest month)	**-0.732648** (*)	0.556718
bio_6a (minimum temperature coldest month)	**-0.959794** (*)	0.175193
bio_7a (temperature annual range)	**0.842822** (*)	0.163407
bio_8a (mean temperature wettest quarter)	**-0.730927** (*)	0.385510
bio_9a (mean temperature driest quarter)	**-0.890100** (*)	0.264902
bio_10a (mean temperature warmest quarter)	**-0.793755** (*)	0.489969
bio_11a (mean temperature coldest quarter)	**-0.956994** (*)	0.229091
bio_12a (annual precipitation)	-0.666655	**-0.706245** (*)
bio_13a (precipitation wettest month)	**-0.701093** (*)	-0.475273
bio_14a (precipitation driest month)	-0.292179	**-0.798126** (*)
bio_15a (precipitation seasonality)	-0.256584	0.603328
bio_16a (precipitation wettest quarter)	-0.699481	-0.512586
bio_17a (precipitation driest quarter)	-0.327346	**-0.806890** (*)
bio_18a (precipitation warmest quarter)	-0.465097	-0.614460
bio_19a (precipitation coldest quarter)	-0.483883	-0.624403
Explained variance	9.840462	5.099666
Proportion of total	46.8593%	24.2841%

## Discussion

*Usnea longissima* is an excellent case study to analyze the causes and consequences of incorrect taxonomic identifications in species occurrence data. The species is generally characterized by its long-pendulous thalli, covering tree branches like garlands, forming cylindrical main branches with vertical, short branchlets resembling fish vertebrae [14, 17, 18, 22]. Many other species resemble *U*. *longissima* in the long-pendulous thalli (see below) and hence have been mistaken for that species; however, morphological, anatomical and chemical details clearly set them apart and make the species readily identifiable by trained lichenologists [14–22].

*Usnea longissima* is very sensitive to environmental changes and is considered an indicator species of well-conserved, humid temperate forest ecosystems, being on the decline or having become extinct from many areas [25–36]. As a consequence, when using historical and modern occurrence data to analyze past and present distributions patterns of such a species, the correct identification of voucher specimens is of critical importance. In the present case, georeferenced GBIF occurrence data would place the species virtually all over the global map and define it as a cosmopolitan taxon. Yet, based on ITS barcoding sequences [17, 24] and regional taxonomic revisions (see below), the species is restricted to the Northern Hemisphere north of the Tropic of Cancer. This is likely a common scenario for other species, particularly among fungi and lichens, where the true ecogeographical distribution of a taxon is often obscured by incorrectly identified collections, either through lack of experience with a group or changing species concept. Similar cases have been reported for other charismatic and presumably widespread macrolichens, such as *Letharia vulpina* in the Parmeliaceae, *Sticta fuliginosa* in the Lobariaceae and *Cora glabrata* in the Hygrophoraceae [43–44, 73–74].

In the case of *Usnea longissima*, existing taxonomic revisions helped to clarify most of the far outliers identified by our predictive niche modeling. Revision of the pendulous species of *Usnea* from Mexico showed that *U*. *longissima* is not present in the area and common misidentifications are based on other long-pendulous species, especially *U*. *angulata* Ach. and *U*. *mexicana* Vain., but also *U*. *ceratina* Ach., *U*. *subgracilis* Vain., and *U*. *transitoria* Motyka [15]. Pendulous species of *Usnea* from Costa Rica were also revised by M. Herrera-Campos (unpubl. data) and the presence of *U*. *longissima* was not confirmed, whereas the report from Colombia represents a previously unrecognized taxon, *U*. *crenulata* Truong & P. Clerc [21]. Other lichens often mistaken for *U*. *longissima* are found in the *Ramalina usnea* complex [75], especially when based on voucherless occurrence data. Molecular phylogenetic revision of pendulous species of *Usnea* from South America further confirmed the absence of *U*. *longissima* from the continent [21]. Taxonomic revision of *Usnea* in Australasia [19–20] also demonstrated the absence of *U*. *longissima*; common misidentifications in the Southern Hemisphere, particularly in the Old World tropics and in Oceania, include *U*. *himantodes* Stirt., *U*. *hossei* Vain., *U*. *misaminensis* (Vain.) Motyka, and *U*. *trichoideoides* Vain. [19–20; P. Clerc, pers. comm. 2014], as well as *U*. *mekista* (Stirt.) D. D. Awasthi. Many of these species were originally described as infraspecific taxa of *U*. *longissima*, emphasizing the likelihood of potential confusion; according to Index Fungorum [76], *U*. *longissima* has over 20 listed infraspecific synonyms.

Thus, among the outliers identified by the predictive model, those from the Neotropics (Mexico to Colombia) and from Australasia (Papua New Guinea, Australia) were confirmed by independent studies to not represent *Usnea longissima*. This leaves occurrence data from New Mexico, the eastern Black Sea area, the northwestern border of Siberia, and the wider Himalaya region for scrutiny. Asian records are in need to be compared to names such as *U*. *hossei*, *U*. *mekista*, *U*. *misaminensis*, and *U*. *trichoideoides*. Since the number of these records is reasonably small (less than 100 compared to a total of 4,672 GBIF records originally analyzed), detailed and targeted taxonomic revision of the vouchers within a short time frame would be feasible. Critical revision of these records would be recommended to assess uncertainties of the model prediction, with the inclusion of subsequently confirmed records representing the species but falling outside the boundaries of the current model. In contrast, revision of the well over 4,000 remaining GBIF records that fall within the narrow model boundaries is not a priority, since even wrongly identified specimens would not affect the model in principle, as they correspond to data points in which *Usnea longissima* occurs or is likely to occur.

Our study thus supports the notion that predictive niche mapping based on confirmed, georeferenced occurrence records is a suitable tool to identify outliers and to considerably narrow down the number of voucher specimens that would require critical revision in order to obtain accurate occurrence data. Obviously, setting up such a study is not trivial, as it requires a large number of confirmed and georeferenced records to be available for a group in question. Ideally, as in the present case [17], such data are directly linked to GenBank sequences, which allows for phylogenetic testing of species concepts. However, modern taxonomic revisions also serve as source for such data, as long as specimen records are georeferenced or can be georeferenced a posteriori. The proposed protocol should work for any organism, as long as its ecological niche can be reasonably well predicted; however, it cannot take into account factors such as human-induced distributions or invasive species, which often occupy different ecological niches in alien ecosystems.

To make best use of strategies to increase the quality of occurrence data, we propose to generate curated specimen data, as already done for fungal ITS barcoding sequence data [1, 48–49]. Since the separate maintenance of such curated databases provides a logistic challenge, a feasible solution would be to annotate occurrence data in existing repositories with a quality score, which indicates whether a particular record has been scrutinized and what the underlying methods were. The type and specimens with sequence data would receive high scores, followed by specimens cited in modern revisions or with annotation labels by a known expert in the field. Additionally, one could implement automated scoring, using high-scored records as templates to highlight unrevised records as likely correct or questionable based on geographic and ecological proximity or distance to high-scored records, including automated background niche modeling [7].

While predictive niche mapping is a useful tool in this context, it also has its limitations. Spatial bias within the sampling area (sampling bias) might lead to false negatives, i.e. sampling points in which the species occurs but has not been sampled, which makes the model boundaries more diffuse. This problem is being addressed by background manipulation and spatial filtering [85–89] and, while sampling bias introduces uncertainties to the boundaries of the model, it will not affect the identification of far outliers, as long as the sampling size is large enough. Spatial bias neglecting areas outside the sampling area (geographical bias) might also generate problems: in the present case, *Usnea longissima* occurs across northern-temperate and boreal forests but only samples from North America and Europe went into the model, disregarding Asia in lieu of georeferenced samples with molecular sequence data. However, the model and the PCA ordination still predicted Asia as part of the theoretical and realized niche. One of the strengths and aims of niche modeling is indeed geographical extrapolation [69–70, 90]; therefore, geographical sampling bias is not necessarily a limitation, as long as the niche is properly represented by sampling size.

The main challenge of niche modeling is the distinction of the potential and the realized niche for the identification of outliers among occurrence data. Since niche mapping is based on environmental parameters, other factors that delimit the realized range of a species, such as ecogeographical barriers, are not taken into consideration. Without these factors, it is impossible to determine whether an occurrence record far outside the known range of a species, but fitting its predicted niche, is a potential misidentification or a range extension. For instance, our model predicts a suitable niche for *Usnea longissima* in Patagonia, and yet the species is absent from South America [21]. Here, we employed PCA ordination of environmental grid parameters and computed the absolute distance to the score representing the optimal set of variables defined by the AUC values to visualize potential barriers. While niche mapping applies a uniformly low score to the area outside the best-fitting grids (blue areas on the heat map), PCA allows to further differentiate the blue area, highlighting areas that are far outside the ecological range of a species. This approach appears promising and could be further enhanced by including estimates of species age and speed of population expansion to compute probability values for potential dispersal over ecogeographical barriers.

## Material and Methods

### Data Sets

We obtained several datasets for this study. First, we downloaded all available ITS barcoding sequences from GenBank labeled *Usnea* (*Dolichousnea*) *longissima*, including as outgroup *U*. *trichodeoides* (Table 2). This included a set of unique haplotypes corresponding to a total of 1,477 samples from 160 locations in North America and Europe [17]. Second, we obtained the corresponding list of the 160 georeferenced locality data for sequenced *Usnea longissima* specimens from the supplemental material of the study by Rolstad et al. [17]. Finally, we downloaded 4,672 georeferenced occurrence records labeled as *U*. *longissima* from GBIF present at the time of accessing the repository. Of these, only 3,950 had valid coordinates (S1 Table), whereas the remaining samples had no values or double zero values in the decimallatitude and decimallongitude fields and were removed from the data set.

**Table 2:** GenBank Accession numbers and voucher information for specimens of *Usnea longissima* used in the phylogenetic and predictice modeling analysis.

Genus	Species	GB Accession	Country	Collector	Number
*Usnea*	*trichodeoides*	AB051665	Japan	Ohmura	2911
*Usnea*	*longissima*	JX978183	Canada	Rolstad et al.	U0001
*Usnea*	*longissima*	JX978184	Canada	Rolstad et al.	U0002
*Usnea*	*longissima*	JX978185	Canada	Rolstad et al.	U0006
*Usnea*	*longissima*	JX978188	Canada	Rolstad et al.	U0039
*Usnea*	*longissima*	JX978189	Canada	Rolstad et al.	U0056
*Usnea*	*longissima*	JX978190	Canada	Rolstad et al.	U0100
*Usnea*	*longissima*	JX978191	Canada	Rolstad et al.	U0116
*Usnea*	*longissima*	JX978192	Canada	Rolstad et al.	U0170
*Usnea*	*longissima*	JX978201	Canada	Rolstad et al.	U0594
*Usnea*	*longissima*	JX978210	Canada	Rolstad et al.	U0918
*Usnea*	*longissima*	KF461130	Canada	McMullin	sn
*Usnea*	*longissima*	AJ748109	Canada	KL	68
*Usnea*	*longissima*	JX978186	USA	Rolstad et al.	U0015
*Usnea*	*longissima*	JX978187	USA	Rolstad et al.	U0035
*Usnea*	*longissima*	JX978193	USA	Rolstad et al.	U0366
*Usnea*	*longissima*	JX978194	USA	Rolstad et al.	U0426
*Usnea*	*longissima*	JX978195	USA	Rolstad et al.	U0437
*Usnea*	*longissima*	JX978197	USA	Rolstad et al.	U0482
*Usnea*	*longissima*	JX978198	USA	Rolstad et al.	U0487
*Usnea*	*longissima*	JX978199	USA	Rolstad et al.	U0551
*Usnea*	*longissima*	JX978200	USA	Rolstad et al.	U0560
*Usnea*	*longissima*	JX978202	USA	Rolstad et al.	U0601
*Usnea*	*longissima*	JX978203	USA	Rolstad et al.	U0657
*Usnea*	*longissima*	JX978204	USA	Rolstad et al.	U0708
*Usnea*	*longissima*	JX978205	USA	Rolstad et al.	U0737
*Usnea*	*longissima*	JX978206	USA	Rolstad et al.	U0742
*Usnea*	*longissima*	JX978207	USA	Rolstad et al.	U0776
*Usnea*	*longissima*	JX978208	USA	Rolstad et al.	U0783
*Usnea*	*longissima*	JX978209	USA	Rolstad et al.	U0841
*Usnea*	*longissima*	JX978211	USA	Rolstad et al.	U1009
*Usnea*	*longissima*	JX978212	USA	Rolstad et al.	U1086
*Usnea*	*longissima*	JX978213	USA	Rolstad et al.	U1590
*Usnea*	*longissima*	JX978214	USA	Rolstad et al.	U1592
*Usnea*	*longissima*	JX978196	Sweden	Rolstad et al.	U0456
*Usnea*	*longissima*	AJ748108	India	KL	88
*Usnea*	*longissima*	DQ383647	SouthKorea	Hur	CH050148
*Usnea*	*longissima*	DQ001304	SouthKorea	Hur	040001
*Usnea*	*longissima*	AB051642	Japan	Ohmura	2877
*Usnea*	*longissima*	AB051643	Japan	Ohmura	2881
*Usnea*	*longissima*	AB051644	Japan	Ohmura	3250
*Usnea*	*longissima*	AB051645	Japan	Ohmura	3664
*Usnea*	*longissima*	AB051646	Japan	Ohmura	3816A
*Usnea*	*longissima*	AB051647	Japan	Ohmura	3816B
*Usnea*	*longissima*	AB051648	Japan	Ohmura	3844
*Usnea*	*longissima*	FJ494936	Taiwan	Shen	L00004685

### Phylogenetic Analysis

ITS barcoding sequences of 46 specimens and unique haplotypes of *U*. *longissima* and one specimen of the outgroup, *U*. *trichodeoides*, were assembled in BioEdit 7.09 [77] and automatically aligned with MAFFT using the—auto option [78]. Unaligned sequences were also subjected to analysis of ambiguously aligned regions using the GUIDANCE webserver [79, 80] and all columns were found to be aligned with high confidence (> 0.95). This resulted in an alignment length of 498 bases. The alignment was subjected to maximum likelihood (ML) search using RAxML 8.0.4 [81], with non-parametric bootstrapping using 1,000 replicates under the universal GTRGAMMA model.

### Predictive Niche Modeling

For the predictive modeling, we used the 160 georeferenced data points [17] corresponding to sequenced specimens of *Usnea longissima* in North America (Alaska: 43; Pacific Northwest: 62; California: 9; Minnesota: 4; Newfoundland and Nova Scotia: 17) and Scandinavia (Norway: 21; Sweden: 4). We employed the bioclim and altitude layers from WorldClim [82] in 2.5 arc minutes (Table 1). *Usnea longissima* in the strict sense as defined here has been reported mainly from old northern-temperate and boreal forest stands [25–36], prompting the inclusion of Global Land Cover Facility land and Landsat Vegetation Continuous Fields (VCF) tree cover layers from GLCF for modeling [83]. To account for spatial sampling bias (false negatives), we applied background manipulation via a bias layer as well as spatial filtering [84–89]. Layers were edited using ArcGIS 10.3 (ERSI). To build ENMs, we used MaxEnt 3.3.3k [90]. For background manipulation of data [84], we ran 100 replicates and withheld 25% of the presence data for testing. To generate 100 spatially filtered datasets, we created a 2x2 degree grid and randomly selected one occurrence from each square in the grid. MaxEnt was then run on each of these datasets and with the same testing parameters. The resulting spatially filtered models were combined to create a composite model. All models were evaluated using the AUC and the Kappa coefficient [91]. While the AUC has been discussed controversely [91], it proved useful for the purpose of the present study.

We used the grids corresponding to the georeferenced occurrence data with the highest AUC values (0.97931) to derive an "optimal" set of bioclim, altitude and land and tree cover layer variables for *Usnea longissima* by computing the median for each parameter from these grids (S1 Table: original grids with AUC values, S2 Table: hypothetical GR_OPTIM with medians). The entire dataset of analyzed grid parameters for the total of 43,967 global grids, including the hypothetical grid, was then subjected to Principal Component Analysis (PCA), extracting two main axes. For both axes, the "optimal" hypothetical grid was used as midpoint and the distance was computed between the midpoint and all other axis scores and then converted into the absolute distance for each grid (S2 Table). The distance values were transformed into color-coded scores and visualized on a global map. While the predictive modeling heatmap only highlights grids based on threshold values, leaving the remainder of the map uniformely blue, PCA ordination visualizes relative "ecological" distances from the optimal niche, thus aiding in detecting potential ecogeographical barriers that would explain differences between the predicted theoretical and and the predicted realized niche.

## Supporting Information

S1 Table List of 3,950 georeferenced GBIF occurrence records labeled *Usnea longissima*. Georeferenced occurrence records contain associated environmental grid data and were used for comparison with the niche model obtained from 160 georeferenced locations in North America and Europe [17], with AUC values indicated.
(XLS)

S2 Table Environmental grid data for the total of 43,967 global grids. Environmental grid data and corresponding raw factor scores derived from PCA analysis for the first and second axis, converted into absolute distance values for each axis (last four columns), together with the hypothecial 'optimal' grid parameters (first row 'GR_OPTIM) derived as medians from all grids with maximum AUC values (0.97931).
(ZIP)
